# Classification of tropical cyclone rain patterns using convolutional autoencoder

**DOI:** 10.1038/s41598-023-50994-5

**Published:** 2024-01-08

**Authors:** Dasol Kim, Corene J. Matyas

**Affiliations:** Department of Geography, University of Florida, Gainesville, FL USA

**Keywords:** Atmospheric science, Natural hazards

## Abstract

Heavy rainfall produced by tropical cyclones (TCs) frequently causes wide-spread damage. TCs have different patterns of rain depending on their development stage, geographical location, and surrounding environmental conditions. However, an objective system for classifying TC rain patterns has not yet been established. This study objectively classifies rain patterns of North Atlantic TCs using a Convolutional Autoencoder (CAE). The CAE is trained with 11,991 images of TC rain rates obtained from satellite precipitation estimates during 2000−2020. The CAE consists of an encoder which compresses the original TC rain image into low-dimensional features and a decoder which reconstructs an image from the compressed features. Then, TC rain images are classified by applying a *k*-means method to the compressed features from the CAE. We identified six TC rain patterns over the North Atlantic and confirmed that they exhibited unique characteristics in their spatial patterns (e.g., area, asymmetry, dispersion) and geographical locations. Furthermore, the characteristics of rain patterns in each cluster were closely related to storm intensity and surrounding environmental conditions of moisture supply, vertical wind shear, and land interaction. This classification of TC rain patterns and further investigations into their evolution and spatial variability can improve forecasts and help mitigate damage from these systems.

## Introduction

Tropical cyclones (TCs) feature winds that spiral towards the low pressure at their center. This converging air uplifts moisture to create clouds which then produce precipitation. Strong uplift forms tall cumulonimbus clouds with high rain rates in their centers surrounded by lower rain rates on the edges^1^. Thinner stratiform clouds form where uplift is weaker and they yield lower rain rates yet can cover a larger area and contribute significantly to the volume of rain^2^. As the organized cloud systems of a TC play an important role in global energy and water balance^3–5^ and may cause serious damages by flooding rains when they move over land^6–10^, it is important to study how their rainbands are organized.

TCs exhibit diverse rainband patterns influenced by various factors such as the speed of the winds within the system and surrounding environmental conditions. In the Atlantic Basin, TC intensity is measured according to the highest 1-min sustained wind speed, which normally occurs near its center. When TCs have high intensity, air will be strongly converged near the storm’s center allowing precipitation to completely wrap around the center and form a round and symmetrical shape^11^. Since the precipitation of TCs originates from water vapor supplied from the warm ocean surface, the strength, area, and total amount of TC rain are significantly affected by the sea surface temperature (SST) and environmental humidity^12–18^. Vertical wind shear refers to a change in wind speed and/or direction with height. When vertical wind shear is strong, the TC vortex tilts in response, promoting precipitation on one side of the storm but limiting it on the other to produce a wavenumber-1 asymmetry^19–26^.

Despite the diversity of spatial patterns in TC rain, a systematic classification method for them has not yet been well developed. Several studies have classified the temporal variations in TC rain rates in fixed regions, but they did not consider the morphological characteristics of the overall rain field of the TC^27–31^. Recently, studies have developed shape metrics using Geographic Information System (GIS) techniques to represent the spatial characteristics of TC rain and classified them accordingly. For example, Matyas^32^ calculated major-to-minor axis ratio, area-to-perimeter, and rain shield arc-length and linked changes in these metrics with weakening of intensity, moisture availability, and terrain after landfall for 13 TCs with radar reflectivity data. Matyas, et al.^33^ renamed rain shield arc-length to closure, and also calculated solidity, dispersion, and fragmentation to compare radar reflectivity values in observed and model-generated TC rain fields. Matyas and Tang^34^ related area, closure, and dispersion of TC radar reflectivity values to TC intensity, TC size, vertical wind shear, and moisture availability. Zhou and Matyas^35^ applied *k*-means clustering to five shape metrics (area, solidity, dispersion, closure, and roundness) of TC rain rates and determined nine regional categories across the North Atlantic. While the clusters they classified mostly represented unique features, some clusters had similar characteristics (e.g., clusters 1 and 2). In addition, there was not a significant difference in certain shape metrics (e.g., roundness) among all clusters. Clustering methods using shape metrics can yield significantly different results depending on the metrics used, and the selection of metrics is subjective, which may make clustering results unstable.

How can we achieve a more objective and robust classification of TC rain patterns? Autoencoders (AEs) are gaining attention as solutions to various image clustering problems. AE is one of the unsupervised deep learning algorithms that compresses input data (i.e., encoding process) and then reconstructs it to be as similar as possible to the original input data (i.e., decoding process)^36^. As the dimension of original data is reduced through the encoding process, clustering methods can be applied including conventional methods (e.g., *k*-means) and embedded clustering layer methods^37–43^. In particular, convolutional autoencoders (CAEs) that utilize convolutional layers can learn the local structure of images, resulting in superior performance in image clustering compared to conventional clustering methods or simple AEs. For example, the classification accuracy of the Modified National Institute of Standards and Technology (MNIST) data, which is a large collection of handwritten digits, is in the range of 80−90% when CAEs were used, while that is only about 55% and 60% when *k*-means and simple AE was used, respectively^44^.

Therefore, we introduce an approach to classify TC rain patterns based on the CAE. Nearly 12,000 rain images from North Atlantic TCs during 2000−2020 are classified, resulting in a finite number of TC rain pattern clusters. This study shows that each of our six clusters exhibit unique characteristics not only in terms of rain patterns but also in development stage of the TC, environmental conditions, and geographical location. Our work represents the first approach in utilizing deep learning for the classification of TC rain patterns. The objective and robust cluster information obtained from this research can serve as fundamental data for enhancing the understanding and forecasting systems of TC rain.

### Convolutional autoencoder (CAE) for TC rain pattern classification

**Figure 1:**
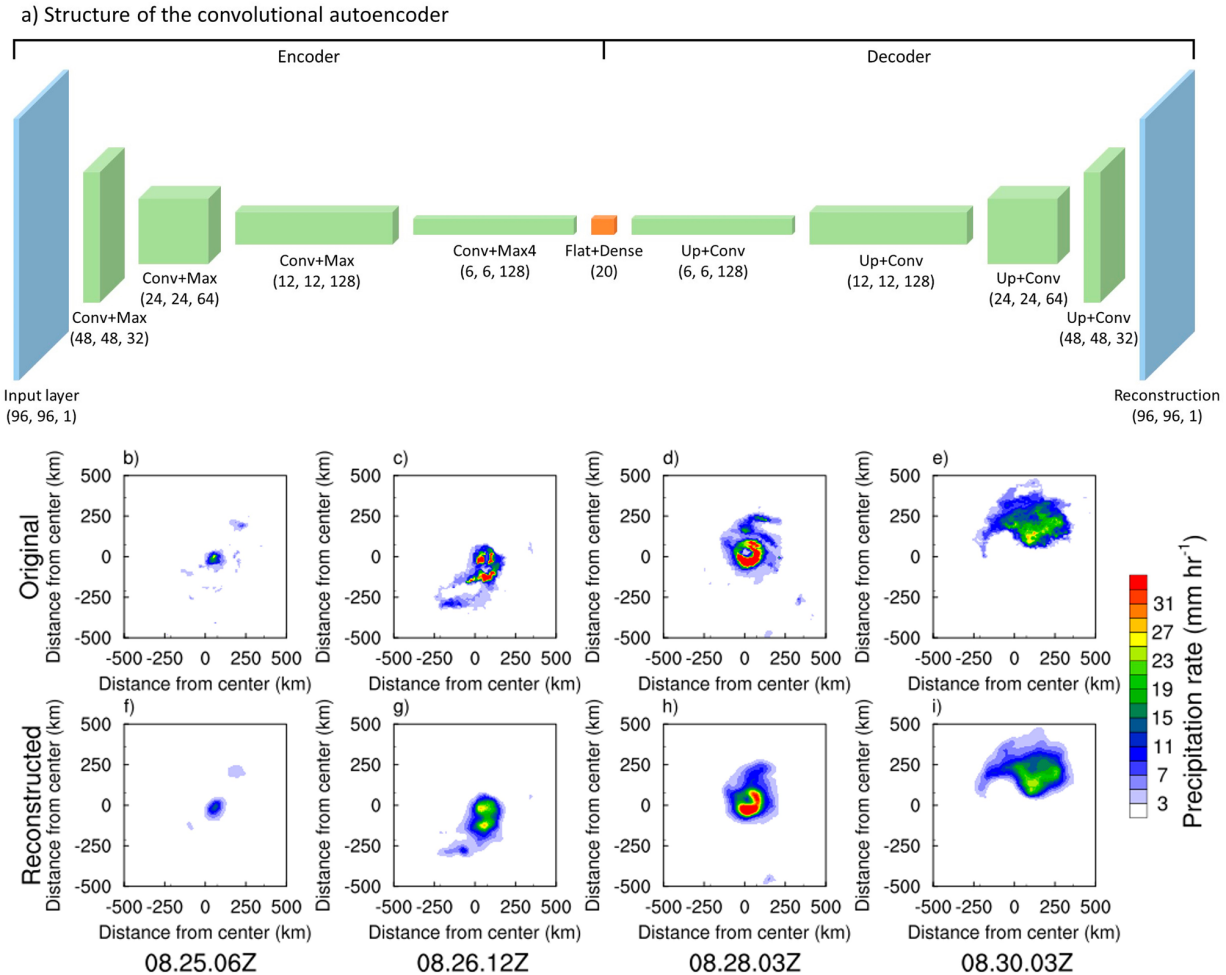
Architecture of the convolutional autoencoder (CAE) model. (**a**) Layer configuration of the CAE. Blue boxes indicate the input and reconstruction layers. Green boxes are convolution plus maxpooling (Conv + Max), and upsampling plus convolution (Up + Conv) layers. Orange box represents the flattening and dense layers. Numbers in parentheses indicate the height, width and depth of each layer. Examples of the (**b**−**e**) original, and (**f**−**i**) reconstructed TC rain images of Hurricane Katrina (2005) at August 25 06Z, 26 12Z, 28 03Z, and 30 03Z, respectively.

A convolutional autoencoder (CAE) in this study consists of an encoder which compresses the original image into low-dimensional features and a decoder which reconstructs the compressed features to an image like the original one (Fig. 1a). The encoder has an input layer and four consecutive convolution and maxpooling (Conv + Max) layers, which are connected to flattening and dense layers (Flat + Dense) with 20 components. In the Conv + Max layers, the width and height decrease by half for each layer, resulting in the original image with a size of 96 × 96 pixels shrinking down to 6 × 6 pixels, while the depth of the layer gradually increases and reaches 128. This process filters and compresses the information that the CAE needs to recognize the image. In the decoder, symmetrically with the encoder, the flattening and dense layers are connected to four consecutive upsampling and convolution (Up + Conv) layers and finally to an output layer having the same dimension as the input layer. The Rectified Linear Unit (ReLU) function is used as the activation function of the CAE except for the last layer where the sigmoid function is used.

The CAE learns features of TC rain images when the decoder reconstructs images similar to the original images given to the encoder. Therefore, the similarity between the original and the reconstructed images is a prerequisite for successful learning of the CAE. Figure 1b−i present examples of original and reconstructed TC rain images of Hurricane Katrina (2005) at different times. The patterns of original and reconstructed images are remarkably similar although there are differences in the area with heavy rain (e.g., red area with rain rate greater than 31 mm hr^−1^ in Fig. 1c vs Fig. 1g). The pattern correlations between the original and reconstructed images range from 0.86 to 0.96 for these examples, which implies that the training of the network was successful.

To evaluate the learning performance of the CAE, we examine the pattern correlation (PCOR), normalized standard deviation (NSTD), mean bias (MB), and root mean squared error (RMSE) between the original and reconstructed images for each TC sample. Here, the NSTD is the ratio of the standard deviation of precipitation in the reconstructed image to that in the original one. Table 1 presents the mean and standard deviation of PCOR, NSTD, MB, and RMSE calculated from all 11,991 samples. The mean PCOR is 0.892, which is markedly higher than results from a simple autoencoder^45^. A NSTD close to 1 means that the variability of precipitation in the original image has been realistically reconstructed and our results are at this high end of the scale. The mean of MB and RMSE are very low, which also indicates a successful outcome. Except for MB, the standard deviation of each variable is considerably smaller than the mean, indicating consistent performance across samples. These results suggest that the CAE learned the features of TC rainfall images well.

**Table 1:** Mean and standard deviation of pattern correlation (PCOR), normalized standard deviation (NSTD), mean bias (MB), and root mean squared error (RMSE) between the entire original and reconstructed TC rainfall images.

	PCOR	NSTD	MB (mm hr^−1^)	RMSE (mm hr^−1^)
Mean	0.892	0.883	0.0003	0.515
Standard deviation	0.072	0.090	0.017	0.207

### Classification of TC rain patterns

**Figure 2:**
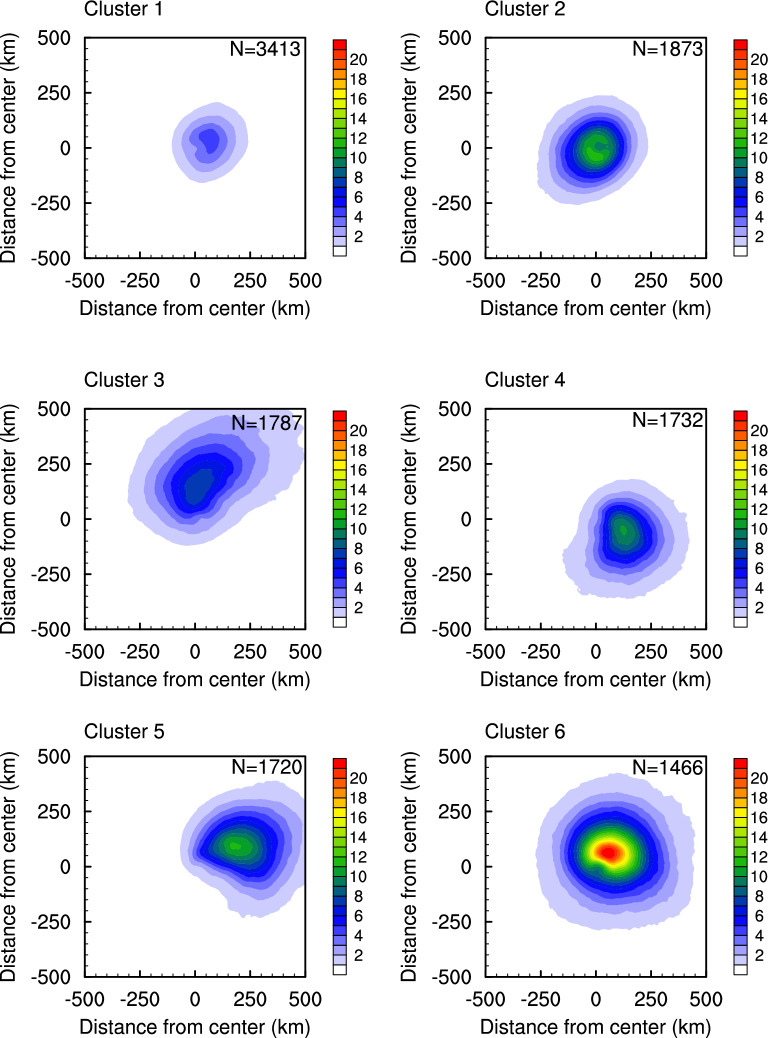
Mean spatial distribution of TC rain (unit: mm hr^−1^) for each cluster. The N at the top-right represents the number of samples in each cluster.

We classify the rain patterns of the North Atlantic TCs into six clusters by applying the *k*-means method to the compressed features of the image obtained from the encoder part of the CAE (Data and Methods). Each cluster presents distinct rain intensity, areal extent, and shape (Fig. 2). For example, the maximum rain rate appears within 50–200 km of TC center in every cluster but is highest in cluster 6 and lowest in cluster 1 (Fig. 2a,e). For the outermost boundary of TC rain, clusters 3 and 6 present larger areas (Fig. 2c,f), while clusters 1 and 2 have smaller areas (Fig. 2a,b). The shape of outermost boundary appears round in clusters 1 and 6, elliptical in clusters 2 and 3, and comma-shaped in clusters 4 and 5. It is notable that cluster 6 has the highest rain rates and is the most compact, but clusters 2, 4, and 5 are not compact despite the considerably high rain rates.

**Figure 3:**
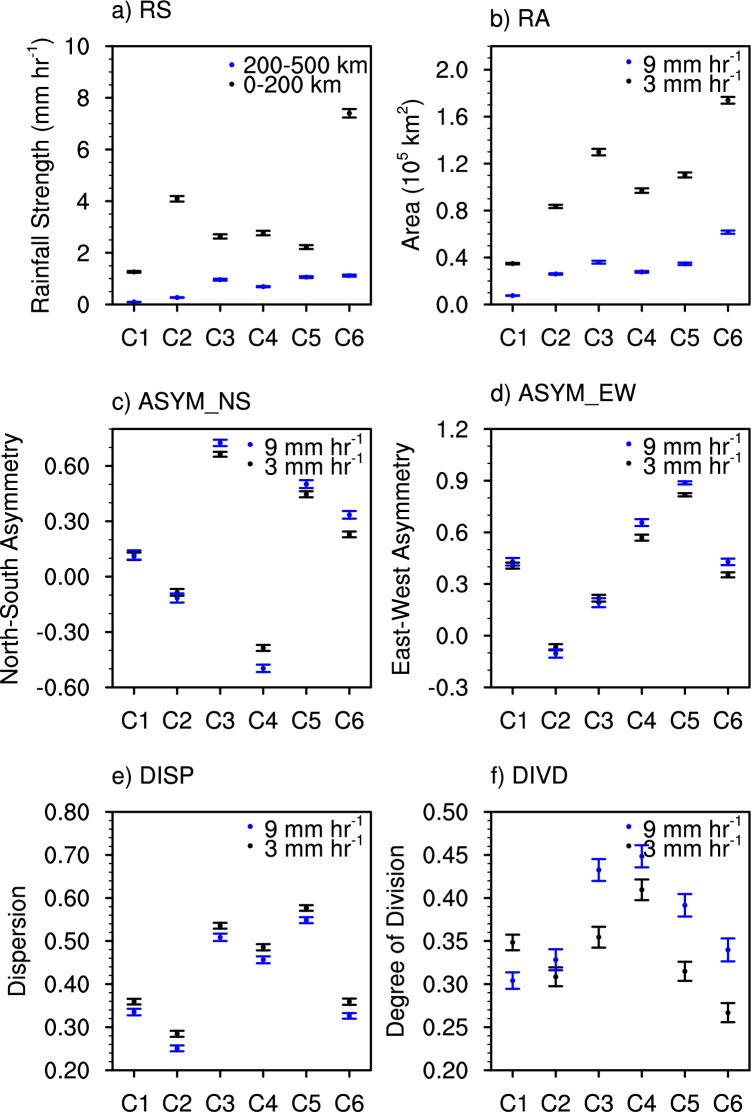
Rainfall strength and shape metrics of each cluster. (**a**) Rainfall Strength (RS), (**b**) Rainfall Area (RA), (**c**) north–south asymmetry (ASYM_NS), (**d**) east–west asymmetry (ASYM_EW), (**e**) dispersion (DISP), and (**f**) degree of division (DIVD) for the six clusters. Dots and error bars denote the mean and 95% confidence interval of the mean, respectively. Black and blue in subplot (**a**) indicate results of RS within 0−200 km radius and within 200−500 km radius, respectively, and those in subplots (**b**−**f**) indicate results of shape metrics for the precipitation criteria of 3 and 9 mm hr^−1^, respectively.

For a more quantitative analysis of the TC rain pattern, mean rainfall strength (RS) within the region of 0–200 and 200–500 km from the storm center (RS200 and RS500, respectively) and five rain shape metrics for each cluster are compared (Fig. 3). The five metrics include rainfall area (RA), asymmetries in north–south and east–west directions (ASYM_NS and ASYM_EW, respectively), dispersion (DISP), and degree of division (DIVD) (Data and methods) which have been commonly used to measure spatial characteristics of TC rain^33,35,46,47^. We calculate the five metrics for pixels with rain rates greater than 3 and 9 mm hr^−1^ to investigate patterns of moderate- and heavy-rain in TCs, respectively. The moderate- and heavy-rain thresholds are based on the sensitivity test on the threshold values of rain rates (Fig. S3). We examined various rain rate thresholds to calculate shape metrics from 1 to 9 mm hr^−1^ with 2 mm hr^−1^ interval. While there are differences in the magnitude of shape metrics in each cluster according to the rain rate threshold (especially in RA), the tendency of variations between clusters remains consistent.

The mean RS200 (rainfall strength in the near-core region) is highest in cluster 6 (7.40 mm hr^−1^) and it is the second highest in cluster 2 (4.09 mm hr^−1^) (Fig. 3a). Note that the mean RS200 of cluster 6 is about six times larger than that of cluster 1 (1.26 mm hr^−1^). There are also significant differences in RS500 (outer rainband region^48^) between clusters, while their variability presents a closer relationship with that of RAs than RS in the core (Fig. 3a,b). The RS500 has correlation coefficient of 0.314, 0.787, and 0.810 with RS200, RA of 3 mm hr^−1^, and RA of 9 mm hr^−1^, respectively, for all samples. This result is consistent with previous studies, indicating that the rainfall in the outer region is closely associated with the total amount and area of TC rain, while its relationship with the intense convection in the core regions is relatively weak^15,16^. Cluster 6 presents the largest mean RA (1.74, and 0.62 × 10^5^ km^2^ for moderate- and heavy-rain, respectively), while cluster 1 has the smallest one (0.35, and 0.08 × 10^5^ km^2^). The heavy rain area is much smaller than moderate rain area, but the variations between clusters are similar in both. Clusters 3, 4, and 5, which have elliptical and comma-shaped patterns are more asymmetrical than other clusters (Fig. 3c,d). The mean ASYM_NS in cluster 3 is the largest (0.66, and 0.72 for moderate- and heavy-rain, respectively), indicating most of rain occurs in the north side of TC. On the other hand, most rain occurs in the east side in cluster 5 with the largest ASYM_EW (0.82, and 0.89). Clusters 3, 4, and 5 also present large DISP, which means the rain appears far from the TC center (Fig. 3e). On the other hand, the clusters 1, 2, and 6 with round rain patterns show small DISP, representing a pattern concentrated in the TC center. Clusters with large DIVD tend to have small RS, except for cluster 4 which has the largest DIVD but moderate RS (Fig. 3f). This implies that the weaker and less developed TCs (i.e., smaller RS) generally have less organized (i.e., more fragmented) rain patterns. The DIVD is also higher in most cases for the heavy-rain (blue color in Fig. 3f) which is expected as areas of convective rain rates can occur in both the inner core and outer rainbands. Only 3 of 180 Mann–Whitney *U* tests retain the null hypothesis that the two clusters have identical median ranks (Table S1). Those three tests are not for the same pair of clusters, which also supports our assertion that the six clusters represent unique distributions of rainfall. Overall, the results indicate that each cluster has a distinct strength and shape which supports our selection of these 6 clusters to represent the range of rain rate patterns existing within Atlantic Basin TCs.

**Figure 4:**
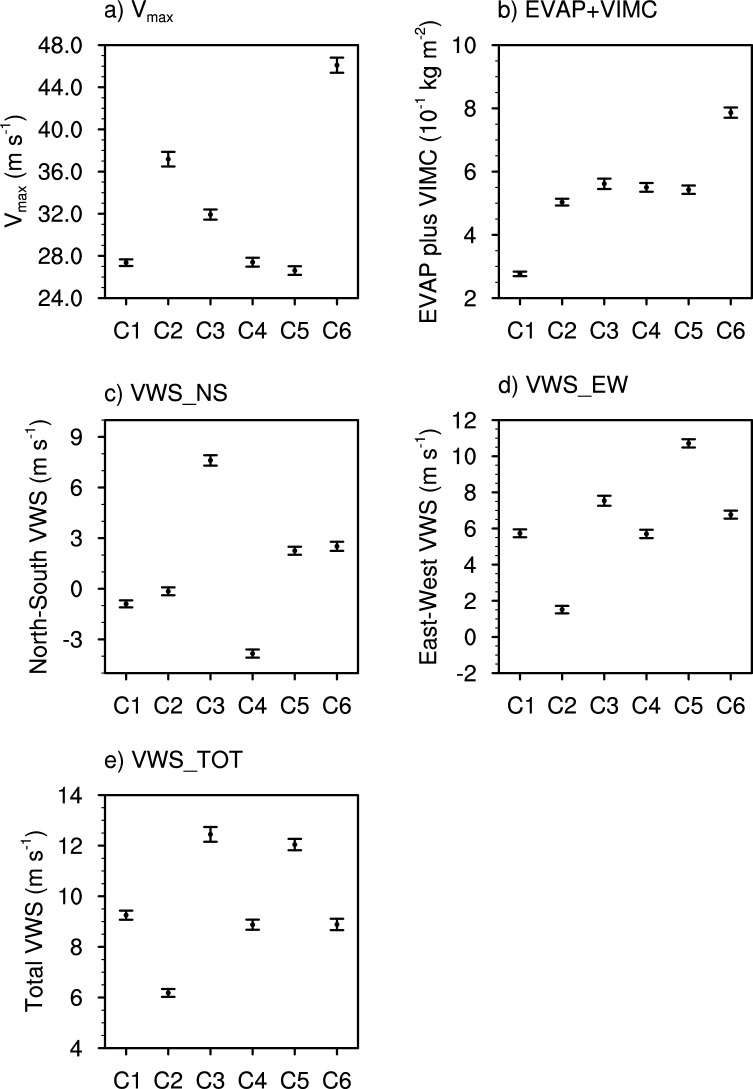
Intensity of TCs, and environmental conditions surrounding TCs of each cluster. (**a**) maximum wind speed (V_max_), (**b**) evaporation plus vertically integrated moisture convergence (EVAP + VIMC), (**c**) north–south vertical wind shear (VWS_NS), (**d**) east–west vertical wind shear (VWS_EW), and (**e**) total vertical wind shear (VWS_TOT) for the six clusters. Dots and error bars denote the mean and 95% confidence interval of the mean, respectively.

Each cluster also exhibits unique conditions in terms of TC intensity and environmental conditions (Data and Methods), which are closely related to the precipitation structure of TCs. Figure 4 presents mean TC intensity and environmental conditions surrounding TCs in each cluster. Spatial patterns of environmental conditions before taking the average around TCs are provided in Figs. S4 and S5. We find a robust relationship between V_max_ and RS200, with the correlation coefficient of 0.53 for all samples. Accordingly, V_max_ is the largest in cluster 6 and the second largest in cluster 2, which corresponds well to the distribution of RS200. Cluster 6 represents the most mature stage of TCs, while cluster 2 appears to be associated with TCs undergoing strong development in a high supply of moisture (evaporation plus vertically integrated moisture convergence; EVAP + VIMC) and weak vertical wind shear (VWS) environment (Fig. 4b–e). Cluster 3 has the third strongest V_max_, but it exhibits a relatively small RS200 given its strong intensity. This can be related to the influence of strong VWS environment (Fig. 4c–e), which causes precipitation to spread asymmetrically rather than concentrating around the center of TC. Clusters 1, 4, and 5 exhibit similar V_max_ values and this similarity is confirmed by the Mann–Whitney *U* tests (Table S2), but RS200 is the smallest in cluster 1 (Fig. 3a vs Fig. 4a). This could be attributed to the significantly drier environment in cluster 1 compared to the other clusters (Fig. 4b and Table S2).

The distribution of the EVAP + VIMC, indicating moisture supply from the environment, is similar to that of RA (Fig. 3b vs Fig. 4b). The EVAP + VIMC has a correlation coefficient of 0.60 and 0.42 with the area of moderate- and heavy-rain, respectively, indicating a close relationship between them. This result is consistent with previous studies suggesting that RA is mainly controlled by environmental moisture and large-scale convection^12–16^. Cluster 3 exhibits a similar value of EVAP + VIMC to clusters 4 and 5 but shows a broader RA. This is associated with the influence of strong VWS in Cluster 3, which causes precipitation to expand asymmetrically and increase in RA^14,18,24,25^. The Mann–Whitney *U* tests confirm that EVAP + VIMC is similar among cases in clusters 3, 4, and 5 (Table S2), however intensity and VWS are significantly different which underscores the importance of examining multiple conditions that can shape a TC’s rainfall production.

The north–south and east–west VWS well-explains the rain asymmetry in each cluster (Fig. 3c vs Fig. 3d and Fig. 4c vs Fig. 4d). In general, TC rain fields extend to the downshear (i.e., the same direction of the shear vector) or downshear-left side (the side to the left of the shear vector)^18–25^. For example, if VWS is eastward (positive VWS_EW), TC rain should be distributed to the east and north. Consistently in our study, the correlation coefficient between the VWS_NS and the ASYM_NS is 0.61 and 0.56 for moderate- and heavy-rain, respectively, and that between the VWS_EW and the ASYM_EW is 0.46 and 0.40 for moderate- and heavy-rain, respectively, indicating the downshear expansion of rain fields. In addition, VWS_EW and ASYM_NS for moderate- and heavy-rain have correlation coefficients of 0.41 and 0.38, which corresponds to the downshear-left asymmetry. Note that most cases feature an eastward component of VWS (Fig. 4d), which should yield positive ASYM_NS and/or ASYM_EW. Figure 3c,d confirm that most cases feature positive ASYM. Similar to VWS_NS and VWS_EW, the VWS_TOT is significantly larger in clusters 3 and 5, supporting the influence of strong vertical wind shear in these clusters (Fig. 4e). Cluster 2 shows the smallest VWS_TOT among others, which might be associated with its relatively compact size but having strong winds and rainfall intensity (Figs. 3a,b and 4a). The Mann–Whitney *U* tests confirm that all six clusters have unique vertical wind shear conditions, except for four out of the 15 cluster combinations. This again supports a six-cluster classification as none of the clusters have the same rainfall strength, shape, TC intensity and environmental conditions.

Previous research demonstrated that VWS, moisture, and storm intensity need to be examined together to better understand the spatial patterns of TC rain rates^13,49^. Our results support their findings as we examine the spatial patterns of moisture (Fig. S4) and VWS (Fig. S5) in conjunction with V_max_ (Fig. 4) to explain the location of highest rain rates (Fig. 2). Clusters 3 and 5 feature asymmetrically-distributed precipitation due to VWS, with the highest rain rates displaced left of shear or downshear. The spatial patterns of moisture also support this displacement as moisture is higher to the right of the shear vector (Fig. S4), with the resulting precipitation located counterclockwise in the direction of the tangential winds. TC intensity (V_max_) is higher for cluster 3, thus the precipitation is advected farther counterclockwise as compared to cluster 5 where intensity is lower. In cluster 6, the semicircle of highest rain rates is also displaced counterclockwise from the region of high moisture. VWS is lower compared to clusters 3 and 5, but V_max_ is much higher suggesting it is the main force driving the displacement.

The variability in rainfall asymmetry among clusters can also be related to the interaction of rain fields with land. Due to greater friction over land compared to oceans, convergence occurs where TC’s winds blow toward land and divergence occurs where winds blow out from land. This effect of land can cause asymmetry in the convection structure within a TC^50,51^. Table S3 provides ratio of samples which are located within 500 km distance from land to the total samples, and ratio of rainfall area over the land to the total rainfall area in each cluster. Half or more of samples in clusters 3, 4, 5, and 6 are located within 500 km distance from land, implying these four clusters likely to be influenced by land. Meanwhile, the ratio of rainfall area over the land is largest in cluster 3 and is in a similar range in other clusters. Therefore, the influence of land may contribute to the large asymmetry in cluster 3.

**Figure 5:**
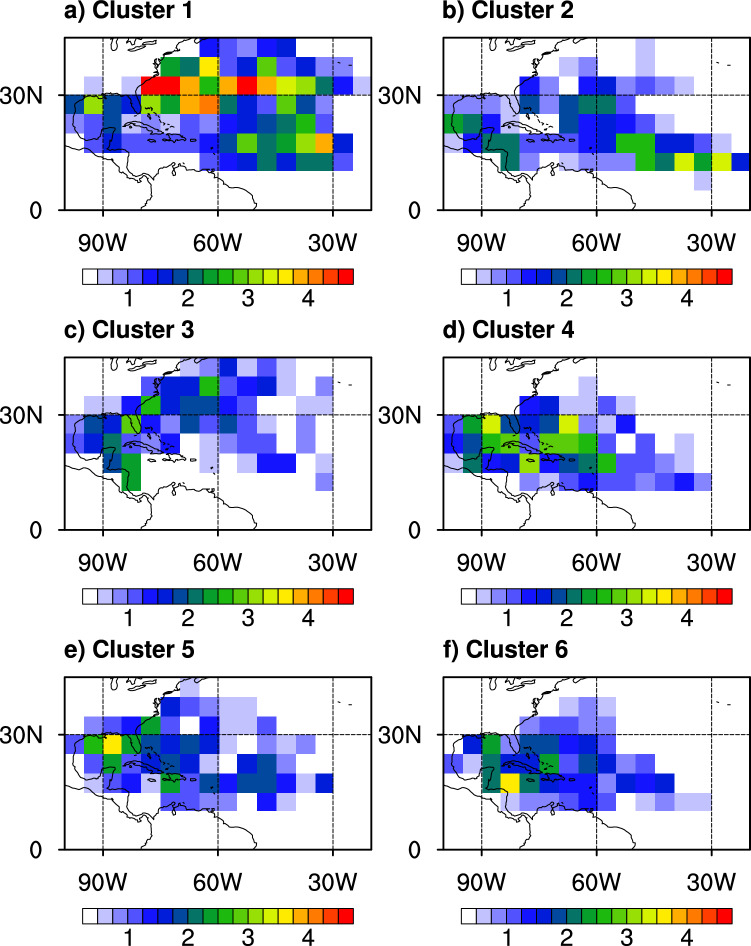
Annual mean occurrence frequency of TC (unit: number year^−1^) for each cluster. The annual occurrence frequency is calculated in each 5° grid box.

Previous studies have shown that regional variations exist in TC rain shape within the Atlantic Basin^35,46^. Thus, we examine the frequency with which each of our clusters occurs over the basin and find distinct variations in the geographical locations of their occurrences. Figure 5 maps the annual mean occurrence frequency of TCs for each cluster. Here, the value of each grid cell represents the annual mean number of TCs appearing in that cell. Cluster 1, which has twice as many samples as the other clusters, shows the widest distribution of occurrences among the six clusters. The annual mean occurrence frequency of cluster 1 is more than two per year in most regions above 25° N and the Main Development Region (MDR^52^) of TCs (10–20° N and 30–55° W). This indicates that cluster 1 includes weak TCs of decaying stage in the mid-latitudes and the early development stage in the MDR where young TCs can entrain dry air from the Saharan Air Layer^53,54^. The large number of samples in cluster 1 is likely because it encompasses both the stages of decaying and early development when TCs produce lower rain rates and are smaller in size. Cluster 2 appears at low latitudes (10–20°N) on the eastern side of the MDR compared to other clusters, which implies that cluster 2 is another mode of TC formation when more moisture is available and vertical wind shear is weaker. These TCs also intensify more quickly as this cluster contains the second highest intensity. Cluster 3 occurs mainly in mid-latitudes where vertical wind shear is known to be strong and near the eastern coast of North America. Cluster 4 appears mainly around the Antilles, while cluster 5 occurs mainly in the Gulf of Mexico. TCs belonging to cluster 6, which has the strongest and widest precipitation, are concentrated in the humid and warm region of northwest Caribbean Sea, farther away from the influence of mid-latitude weather systems than cluster 5.

## Discussion and conclusion

Understanding various rain patterns of TCs is essential for comprehending the nature of TCs and improving prediction of their rainfall. However, there is still a lack of well-developed systems for objectively classifying TC rain patterns. This study utilized a Convolutional Autoencoder (CAE) to classify the rain patterns of North Atlantic TCs. We determined that six clusters presented the optimal number to demonstrate distinct characteristics in rain pattern, development stage of the TC, environmental conditions, and geographical location. Cluster 1, which had the weakest and narrowest precipitation among all clusters as well as having twice the number of samples, included weak TCs in the mid-latitudes where many TCs dissipate or lose their tropical characteristics and farther south in the main development region (MDR). Cluster 2 showed stronger precipitation within a smaller area compared to other clusters and primarily appeared in the low latitudes, including the MDR. Although both clusters 1 and 2 included TCs developing in the MDR, there were contrasting environmental conditions as cluster 2 TCs were stronger, in a moister environment, and experienced less vertical wind shear as compared to cluster 1. Cluster 3 had the greatest north–south asymmetry and the second-largest rain area. It had the strongest north–south vertical wind shear and occurred most frequently off the eastern coast of North America in both subtropical and middle latitudes. Clusters 4 and 5 exhibited comma-shaped rain patterns and showed significant asymmetry towards the southeast and east, respectively, influenced by vertical wind shear. Although they both occurred over the Gulf of Mexico, this location was dominant for cluster 5 while cluster 4 occurred in equal frequency east and north of the Antilles. Cluster 6 represented mature TCs, displaying small asymmetry and the strongest and widest precipitation among all clusters. Cluster 6 primarily appeared in the northwest region of the Caribbean Sea, which had the highest moisture supply.

Our research is the first attempt to classify the spatial patterns of TC rain using deep learning. Previous studies have used several shape metrics that can represent the characteristics of TC rain patterns for classification. However, a limitation of previous studies was that the clustering results could vary significantly depending on the subjective selection of shape metrics by the researchers. The CAE overcame this limitation and demonstrated more objective clustering results with clear differences between clusters. Regarding the track patterns of TCs, many previous studies have classified them and revealed the mechanisms that regulate their occurrences^55–58^. Furthermore, previous studies have developed models to predict the occurrences of each track cluster and applied them to season forecasting of TC activity^59–61^. Similarly, the six clusters identified through the CAE can provide fundamental information for understanding the mechanisms regulating TC rain and improving forecasting skills. Additionally, utilizing well-trained CAE enables fast classification of numerous images even with limited computing resources. Therefore, the CAE can be effectively applied to classify TC rain patterns in various data sources such as radar, reanalysis, and numerical forecast data.

Our future analysis will utilize the CAE to examine spatial patterns of TC rain across all ocean basins where TCs exist. We aim to uncover patterns unique to each basin given their different distributions of land and ranges of latitudes with suitable ocean conditions and access to deep tropical moisture to support TCs as well as determine whether common patterns exist across the globe (e.g., high rain strength and symmetry with low dispersion for highly intense systems). Given that IMERG data are available for more than 20 years, we also aim to look at changes in rain patterns over time as increasing moisture availability could lead to greater rainfall strength, larger rainfall area, or a combination of both and possibly help TCs maintain high rain rates despite higher amounts of vertical wind shear.

## Data and methods

### Data

Information of North Atlantic TCs during 2000–2020 was obtained from the International Best Track Archive for Climate Stewardship (IBTrACS^62^) version 4, including the time, location of TC center in latitude and longitude, 1-min averaged maximum sustained wind speed (V_max_), radius of 17 m s^−1^ (R17) in four quadrants and type of storm in 3-h intervals. The V_max_ indicates the intensity of a TC and is known as a primary factor controlling rain rate near the TC center since faster winds indicate a stronger circulation to converge and uplift moisture to produce precipitation^13–17,26^. In the North Atlantic, when V_max_ reaches 17 m s^−1^, a TC is termed a tropical storm and at 33 m s^−1^, a hurricane. Using storm type information and V_max_, only tropical storms and hurricanes with intensity of greater than 17 m s^−1^ were included in this study. Global precipitation data were collected from the Global Precipitation Measurement (GPM) IMERG V06 (Integrated Multi-Satellite Retrievals for GPM, version 06)^63^. The spatial and temporal resolutions of the IMERG data are 0.1° and 30-min, respectively. As the IMERG precipitation data are provided from 2000, the analysis period starts from 2000.

The environmental conditions analyzed in this study included evaporation (EVAP), vertically integrated moisture convergence over the entire atmospheric column (VIMC), and vertical wind shear between 200 and 850 hPa levels in a north–south (VWS_NS) and east–west (VWS_EW) directions. These environmental variables were collected using the 5th generation reanalysis data from the European Centre for Medium-Range Weather Forecasts (ERA5)^64^. The land-sea mask data are obtained from the ERA5 to investigate the ratio of rainfall area over the land. Pixels with a land-sea mask value greater than 0.5 were treated as land, otherwise they were treated as ocean. The ERA5 reanalysis data are provided with a horizontal and temporal resolution of 0.25° and 1-h, respectively.

### Environmental conditions surrounding tropical cyclones

The moisture supplied to the TC from the surrounding environmental conditions originates from evaporation (EVAP) from warm ocean surfaces and moisture convergence in the troposphere (vertically integrated moisture convergence; VIMC)^65^. Thus, the environmental moisture supply was defined as the sum of EVAP and VIMC. Vertical wind shear (VWS), the difference in winds between two altitudes, was analyzed for the north–south (VWS_NS) and east–west (VWS_EW) components (Eqs. 1 and 2).

1 $$VW{S}_{-}NS = {v}_{200} - {v}_{850}$$

2 $$VW{S}_{-}EW = {u}_{200} - {u}_{850}$$

where ${v}_{200}$ and ${v}_{850}$ are the north–south component of wind at 200 and 850 hPa altitudes, respectively. Similarly, ${u}_{200}$ and ${u}_{850}$ represent the east–west component of wind at 200 and 850 hPa altitudes, respectively. Thus, VWS_NS and VWS_EW are positive when the shear is southerly and westerly, respectively. The total vertical wind shear (VWS_TOT) is defined as $\sqrt{{VWS\_NS}^{2}+{VWS\_EW}^{2}}$. The environmental conditions were averaged within 800 km radius from the storm center. For the calculation of VWS, values within 200 km of each TC sample were not analyzed to exclude strong circulation of the TC^15,16,24,34,46^. As the R17 can represent the spatial scale of major vortex of a TC, we also performed the analyses using R17. However, given that the average value for R17 (186 km) is close to 200 km, the results did not show significant differences when using each TC’s R17 compared to applying a consistent 200 km radius. Thus, we only present results with the 200 km radius.

### Pre-processing

TC rain images were cropped from the IMERG precipitation data in a grid box of 96 × 96 pixels (approximately 1000 km × 1000 km size) along the TC center. Since the cropped image may contain rain fields not associated with the TC, only TC rain fields were extracted using the following method: 1) all rain polygons were searched in the cropped image where a rain polygon is defined as a set of one or more connected pixels with rain rates ≥ 1 mm h^−1^, 2) rain polygons where the minimum distance between the edge of rain polygon and the TC center is > 500 km were removed^14,35,46,66,67^. This study focuses on major rain field of TC within 500 km radius including the eyewall and the rain band structures that spirals into the storm center^1,68^. Then, rain rates in the images were normalized to have a range from 0 to 1 using a min–max method. TC center was provided from the IBTrACS data, and TC rain images were also obtained every 3-h. The total number of TC rain images is 11,991 from 336 TC cases during the analysis period (2000–2020).

### Shape metrics

Characteristics of TC rain patterns were investigated based on the rainfall strength and five shape metrics which have been widely used in previous studies. The rainfall strength (RS), which is the mean precipitation within specific radius, is originally defined within 200 km radius from the TC center including pixels with a rain rate of 0 mm hr^−1^ to represent magnitudes of TC rain in the near-core region of the storm^15,16^. In this study, the RS was defined within the region of 0–200 and 200–500 km radius (RS200 and RS500, respectively) to examine magnitudes of rain in the near-core and the outer region of TC, respectively. We selected 200 km as the boundary between the near-core region of the storm and outer region rainbands as it corresponds well to the quadrant-averaged R17 which had a mean of 186 km. The rainfall area (RA) is the total area of the TC rain field, indicating the spatial scale of the TC rain. Asymmetries of TC rain in the north–south and east–west directions (ASYM_NS, and ASYM_EW) are defined as the Eqs. (3) and (4).

3 $$ASY{M}_{-}NS = \frac{R{A}_{north} - R{A}_{south}}{RA}$$

4 $$ASY{M}_{-}EW = \frac{R{A}_{east} - R{A}_{west}}{RA}$$

where $R{A}_{north}$, $R{A}_{south}$, $R{A}_{east}$, and $R{A}_{west}$ are areas of rain field in the north, south, east, and west side of TC center, respectively. The direction for a positive result is set to match with the hypothesized relationship between the direction of VWS and subsequent displacement of rainfall in downshear direction (the same direction of the shear vector). Dispersion (DISP) indicates the spread of the centers of rain fields away from the TC center and defined as the Eq. (5).

5 $$DISP = {\sum }_{i=1}^{N}\frac{R{A}_{i}}{RA}\frac{{r}_{centroid, i}}{{r}_{search}}$$

where $R{A}_{i}$ and ${r}_{centroid, i}$ are the area and the centroid radius of ith rain polygon, respectively, ${r}_{search}$ is the search radius (500 km) of rain polygon^35,47^. Larger polygons receive higher weights in the calculation. Degree of division (DIVD)^69^ measures the fragmentation of rain field, which is defined as the Eq. (6).

6 $$DIVD = 1 - {\sum }_{i=1}^{N}{\left(\frac{R{A}_{i}}{RA}\right)}^{2}$$

### Clustering using *k*-means

To extract information about the spatial patterns presented within TC rainbands, we applied the *k*-means clustering method to the flattened dense layer (i.e., the vector of 20 components; orange box in Fig. 1a) obtained from the encoder part of CAE and containing the compressed features of original images. The *k*-means method is an unsupervised learning algorithm that forms clusters based on the similarity among dataset^70^. Applying the *k*-means method directly to the original images with hundreds or thousands of components (e.g., 96 × 96 pixels in this study) can be challenging due to the high dimensionality, which can hinder proper classification. However, applying *k*-means to the small number of features compressed by the CAE can significantly improve the classification performance^44^. In other words, the CAE plays a crucial role in reducing the dimensionality of the original image, which enables the application of *k*-means clustering.

The number of clusters *k* was determined based on the total within-cluster sum of squares (WSS) and the elbow method^35,71^. Figure S1 illustrates the total WSS for *k* ranging from 2 to 15 and the difference of WSS from *k* − 1 to *k*. As the *k* increases, the total WWS decreases. Since WWS represents the variability within clusters, a smaller total WWS indicates higher compactness of the cluster. Generally, a smaller total WWS can be interpreted as better classification. However, if the number of clusters becomes too large, the differences between clusters may not be meaningful (e.g., when one cluster with similar properties is divided into two). Therefore, the point where the curve of WWS difference (red line in Fig. S1) changes abruptly, known as the "elbow," can be defined as the optimal number of clusters. According to the elbow method, the optimal cluster number for Atlantic Basin TC rain patterns is between 5 and 7. We tested the clustering results by varying *k*, the number of clusters, from 4 to 8 (Fig. S2). When the *k* increased by one from 4 to 6, clusters with distinct characteristics were added. For example, when the 4-cluster solution expands to 5-clusters, cluster 3 subdivides into TCs with more rain west of center (new cluster 3) and east of center (new cluster 4) while old cluster 4 shows increased rain rates overall as it becomes new cluster 5. This cluster then remains remarkably similar despite more clusters being added. When the number reached 7 and higher, clusters with similar characteristics were divided into multiple clusters. For example, when comparing k = 6 and k = 7, cluster 3 subdivides into clusters 3 and 4. Our assertion that 6 is the optimal number of clusters is further supported by the spatial and environmental analyses described in the main text.

### Statistical analysis

We performed non-parametric Mann–Whitney *U* tests to compare clusters in pairs to determine the likelihood that each pair is from the same distribution. T﻿he data in each cluster were ranked and the ranks were then compared between each cluster pair. The null hypothesis was that the two clusters have identical medians. The test was performed with α = 0.05 and the null hypothesis was rejected when *p* < 0.05, which supported our assertion that the clusters represent distinct spatial distributions of TC rain rates. We performed 165 of these tests considering the 11 measures of rainfall distribution with 15 pairings of each of the 6 clusters. We also performed tests for intensity, moisture, and VWS over the 15 pairs.

### Supplementary Information

Supplementary Information.

## Data Availability

The IBTrACS version 4 data are available at https://www.ncei.noaa.gov/data/international-best-track-archive-for-climate-stewardship-ibtracs/v04r00/access/netcdf/. The IMERG precipitation data can be downloaded at https://disc.gsfc.nasa.gov/datasets/GPM_3IMERGHH_06/summary?keywords=IMERG. The ERA-5 reanalysis data can be obtained from https://cds.climate.copernicus.eu/#!/search?text=ERA5&type=dataset. The shape metrics calculated from the IMERG precipitation data are available from the corresponding author upon reasonable request.

## Code availability

The source code for the convolutional autoencoder is available from the corresponding author upon reasonable request.
