# Supplementary material for: Classification of tropical cyclone rain patterns using convolutional autoencoder
Source: Sci Rep. 2024 Jan 8;14:791. doi: 10.1038/s41598-023-50994-5 (PMC10774432; doi:10.1038/s41598-023-50994-5)
Supplement: Supplementary file 1 — Supplementary Information. [file 41598_2023_50994_MOESM1_ESM.docx]

Supplementary information for classification of tropical cyclone rain patterns using convolutional autoencoder

Dasol Kim^1^, and Corene J. Matyas^1,*^

^1^Department of Geography, University of Florida, Gainesville, Florida, USA

Scientific Reports

*Correspondence to: Corene J. Matyas, matyas@ufl.edu

Table S1. The p-value for the population difference in the rainfall strength and five shape metrics between clusters. The results of the five shape metrics are for moderate (3 mm hr^-1^) and heavy (9 mm hr^-1^) rain. The bold font indicates that the null hypothesis (no difference between the two groups' populations) was accepted at the 95% confidence level.

| Clusters | RS | | RA | | ASYM_NS | | ASYM_EW | | DISP | | DIVD | |
| --- | --- | --- | --- | --- | --- | --- | --- | --- | --- | --- | --- | --- |
|  | 0–200 km | 200–500 km | 3 mm hr^-1^ | 9 mm hr^-1^ | 3 mm hr^-1^ | 9 mm hr^-1^ | 3 mm hr^-1^ | 9 mm hr^-1^ | 3 mm hr^-1^ | 9 mm hr^-1^ | 3 mm hr^-1^ | 9 mm hr^-1^ |
| 1 vs 2 | < 0.001 | < 0.001 | < 0.001 | < 0.001 | < 0.001 | < 0.001 | < 0.001 | < 0.001 | < 0.001 | < 0.001 | < 0.001 | < 0.001 |
| 1 vs 3 | < 0.001 | < 0.001 | < 0.001 | < 0.001 | < 0.001 | < 0.001 | < 0.001 | < 0.001 | < 0.001 | < 0.001 | 0.020 | < 0.001 |
| 1 vs 4 | < 0.001 | < 0.001 | < 0.001 | < 0.001 | < 0.001 | < 0.001 | < 0.001 | < 0.001 | < 0.001 | < 0.001 | < 0.001 | < 0.001 |
| 1 vs 5 | < 0.001 | < 0.001 | < 0.001 | < 0.001 | < 0.001 | < 0.001 | < 0.001 | < 0.001 | < 0.001 | < 0.001 | 0.006 | < 0.001 |
| 1 vs 6 | < 0.001 | < 0.001 | < 0.001 | < 0.001 | < 0.001 | < 0.001 | < 0.001 | < 0.001 | < 0.001 | < 0.001 | < 0.001 | < 0.001 |
| 2 vs 3 | < 0.001 | < 0.001 | < 0.001 | < 0.001 | < 0.001 | < 0.001 | < 0.001 | < 0.001 | < 0.001 | < 0.001 | < 0.001 | < 0.001 |
| 2 vs 4 | < 0.001 | < 0.001 | < 0.001 | 0.002 | < 0.001 | < 0.001 | < 0.001 | < 0.001 | < 0.001 | < 0.001 | < 0.001 | < 0.001 |
| 2 vs 5 | < 0.001 | < 0.001 | < 0.001 | < 0.001 | < 0.001 | < 0.001 | < 0.001 | < 0.001 | < 0.001 | < 0.001 | **0.094** | < 0.001 |
| 2 vs 6 | < 0.001 | < 0.001 | < 0.001 | < 0.001 | < 0.001 | < 0.001 | < 0.001 | < 0.001 | < 0.001 | < 0.001 | < 0.001 | 0.018 |
| 3 vs 4 | 0.018 | < 0.001 | < 0.001 | < 0.001 | < 0.001 | < 0.001 | < 0.001 | < 0.001 | < 0.001 | < 0.001 | < 0.001 | 0.038 |
| 3 vs 5 | < 0.001 | < 0.001 | < 0.001 | **0.121** | < 0.001 | < 0.001 | < 0.001 | < 0.001 | < 0.001 | < 0.001 | < 0.001 | < 0.001 |
| 3 vs 6 | < 0.001 | < 0.001 | < 0.001 | < 0.001 | < 0.001 | < 0.001 | < 0.001 | < 0.001 | < 0.001 | < 0.001 | < 0.001 | < 0.001 |
| 4 vs 5 | < 0.001 | < 0.001 | < 0.001 | < 0.001 | < 0.001 | < 0.001 | < 0.001 | < 0.001 | < 0.001 | < 0.001 | < 0.001 | < 0.001 |
| 4 vs 6 | < 0.001 | < 0.001 | < 0.001 | < 0.001 | < 0.001 | < 0.001 | < 0.001 | < 0.001 | < 0.001 | < 0.001 | < 0.001 | < 0.001 |
| 5 vs 6 | < 0.001 | **0.124** | < 0.001 | < 0.001 | < 0.001 | < 0.001 | < 0.001 | < 0.001 | < 0.001 | < 0.001 | < 0.001 | < 0.001 |

Table S2. The p-value for the population difference in the TC intensity, and environmental conditions between clusters. The bold font indicates that the null hypothesis (no difference between the two groups' populations) was accepted at the 95% confidence level.

| Clusters | Vmax | EVAP+VIMC | VWS_NS | VWS_EW | VWS_TOT |
| --- | --- | --- | --- | --- | --- |
| 1 vs 2 | < 0.001 | < 0.001 | < 0.001 | < 0.001 | < 0.001 |
| 1 vs 3 | < 0.001 | < 0.001 | < 0.001 | < 0.001 | < 0.001 |
| 1 vs 4 | **0.099** | < 0.001 | < 0.001 | **0.346** | **0.276** |
| 1 vs 5 | **0.074** | < 0.001 | < 0.001 | < 0.001 | < 0.001 |
| 1 vs 6 | < 0.001 | < 0.001 | < 0.001 | < 0.001 | **0.211** |
| 2 vs 3 | < 0.001 | < 0.001 | < 0.001 | < 0.001 | < 0.001 |
| 2 vs 4 | < 0.001 | < 0.001 | < 0.001 | < 0.001 | < 0.001 |
| 2 vs 5 | < 0.001 | < 0.001 | < 0.001 | < 0.001 | < 0.001 |
| 2 vs 6 | < 0.001 | < 0.001 | < 0.001 | < 0.001 | < 0.001 |
| 3 vs 4 | < 0.001 | **0.110** | < 0.001 | < 0.001 | < 0.001 |
| 3 vs 5 | < 0.001 | 0.023 | < 0.001 | < 0.001 | **0.280** |
| 3 vs 6 | < 0.001 | < 0.001 | < 0.001 | < 0.001 | < 0.001 |
| 4 vs 5 | 0.005 | **0.198** | < 0.001 | < 0.001 | < 0.001 |
| 4 vs 6 | < 0.001 | < 0.001 | < 0.001 | < 0.001 | **0.416** |
| 5 vs 6 | < 0.001 | < 0.001 | **0.390** | < 0.001 | < 0.001 |

Table S3. Ratio of samples within 500 km distance from land to the total samples, and ratio of rain area over land for each cluster. The ratio of rain area over land is calculated for only the samples within 500 km distance from land.

|  | Ratio of samples within 500 km distance from land (%) | Ratio of rain area over land (3 mm hr^-1^) | | Ratio of rain area over land (9 mm hr^-1^) | | |
| --- | --- | --- | --- | --- | --- | --- |
|  |  | Mean (%) | Standard deviation (%) | Mean (%) | Standard deviation (%) | |
| Cluster 1 | 29.8 | 16.0 | 25.7 | 14.6 | | 27.9 |
| Cluster 2 | 37.6 | 17.5 | 23.8 | 14.6 | | 25.0 |
| Cluster 3 | 49.7 | 28.4 | 31.3 | 26.3 | | 34.0 |
| Cluster 4 | 57.0 | 15.4 | 19.4 | 11.8 | | 19.5 |
| Cluster 5 | 55.8 | 16.5 | 24.3 | 13.8 | | 25.3 |
| Cluster 6 | 56.3 | 13.1 | 19.7 | 10.3 | | 21.1 |


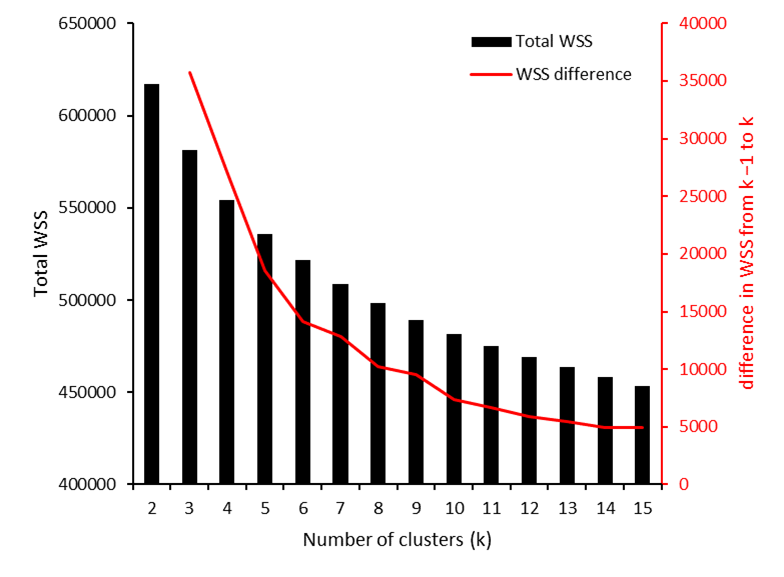


Fig. S1. The total within-cluster sum of squares (WSS) according to the number of clusters *k* (black bars) and its difference from *k*−1 to *k* (red line).


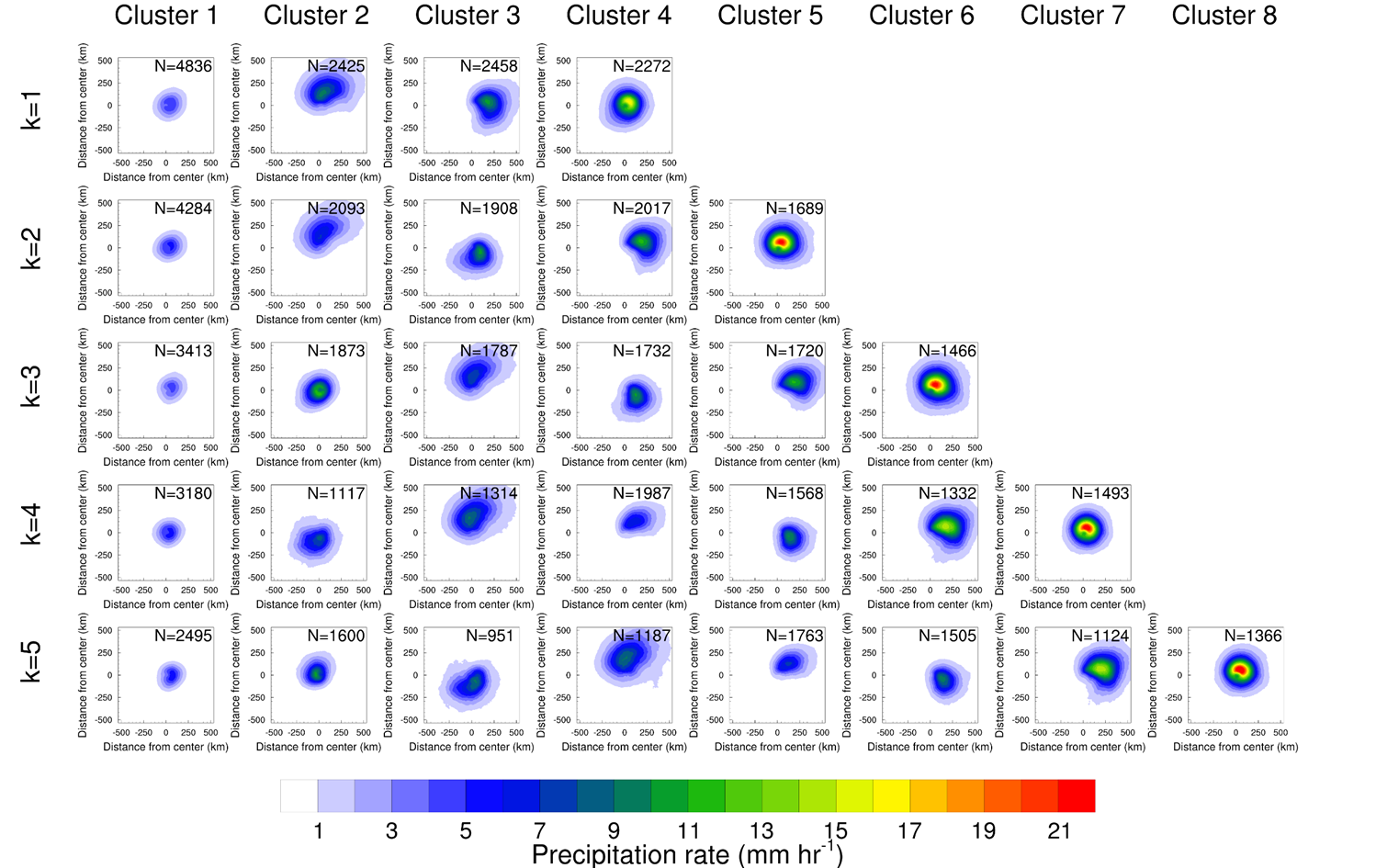


Fig. S2. Mean spatial distributions of TC rain (unit: mm hr^-1^) for cluster solutions 4 to 8.

**
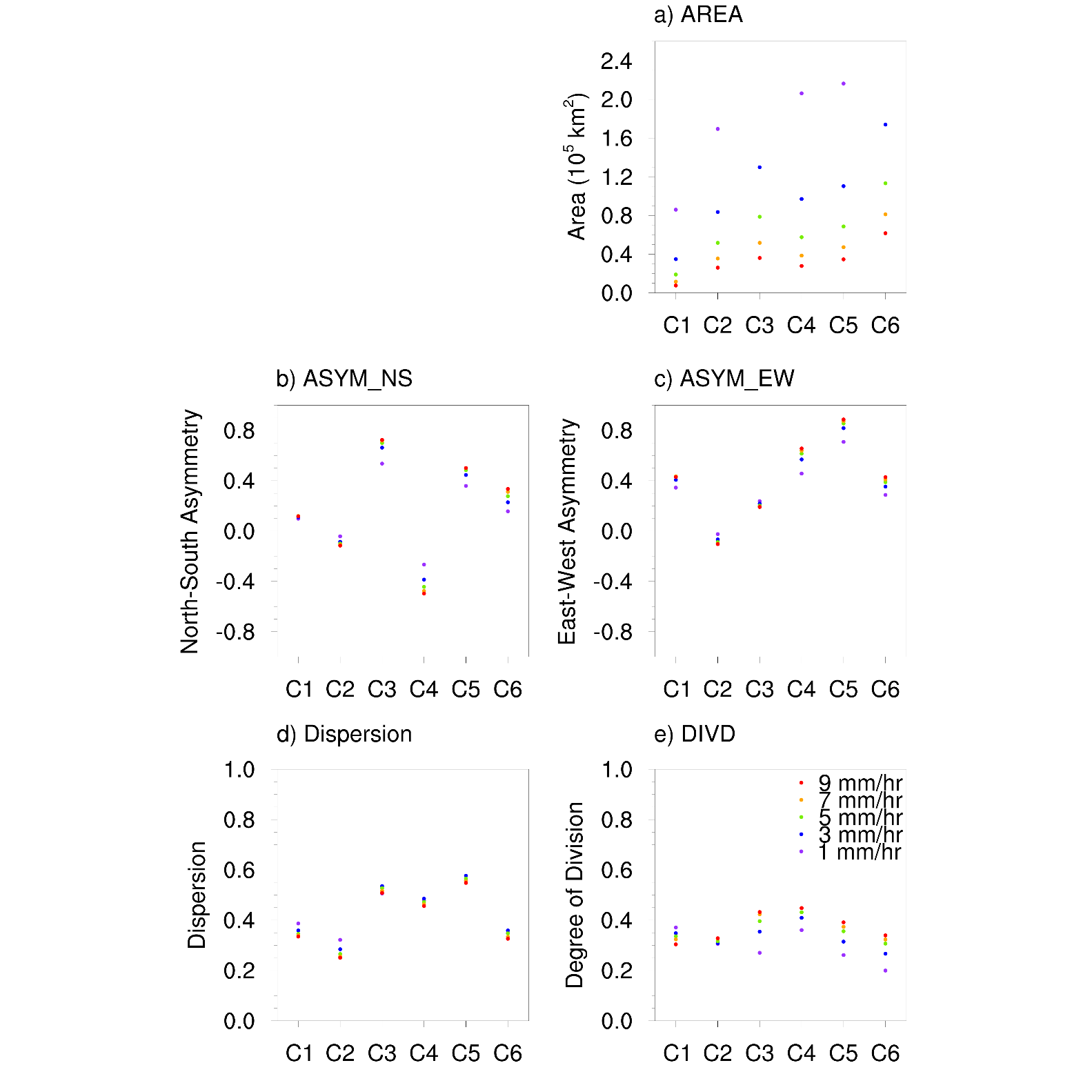
**

Fig. S3. Mean rainfall shape metrics for precipitation criteria of 1, 3, 5, 7, and 9 mm hr^-1^ (red, orang, green, blue, purple, respectively). a, Rainfall Area (RA), b, north-south asymmetry (ASYM_NS), c, east-west asymmetry (ASYM_EW), d, dispersion (DISP), and e, degree of division (DIVD) for the six clusters. Dots and error bars denote the mean and 95% confidence interval of the mean, respectively.


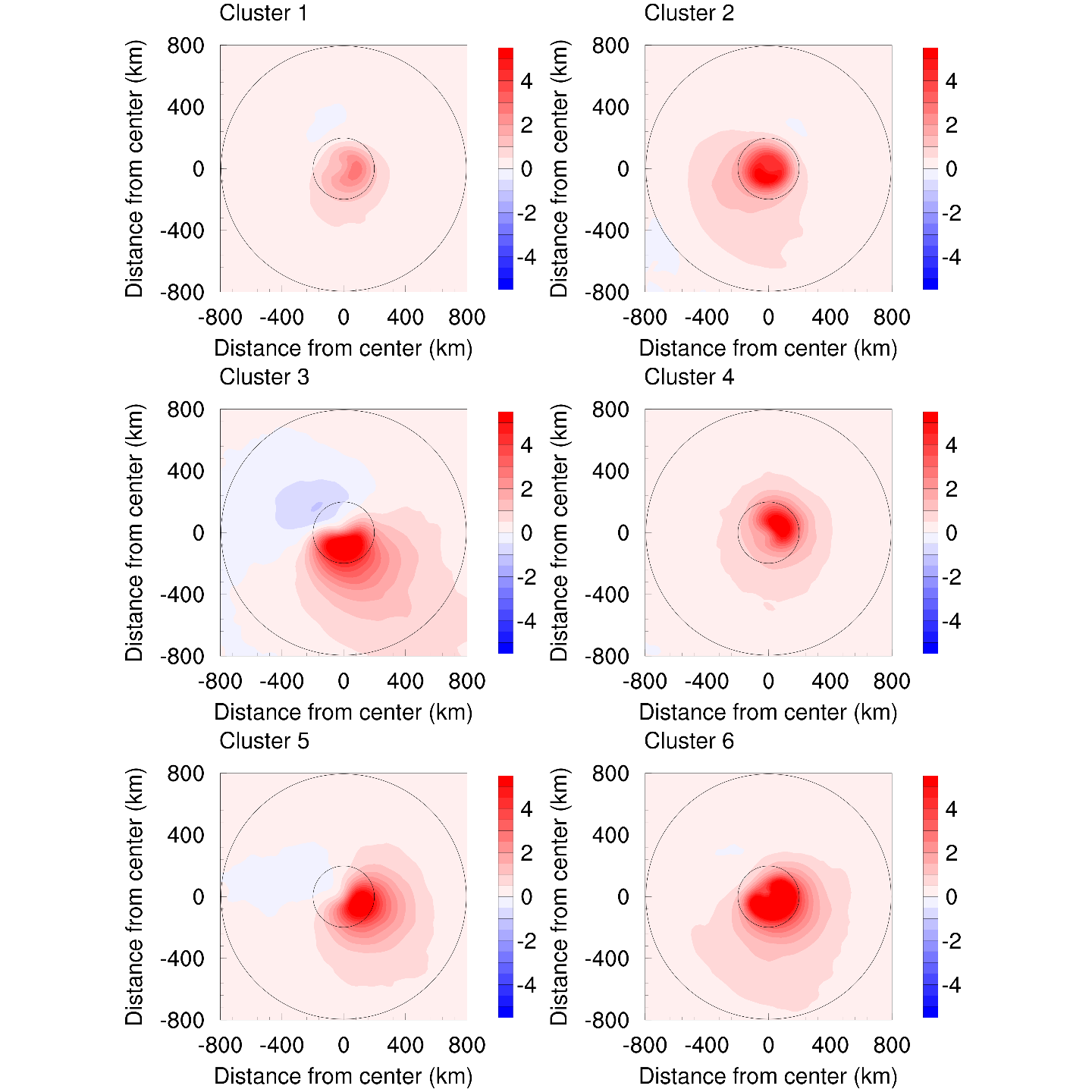


Fig. S4. Mean spatial distribution of evaporation plus vertically integrated moisture convergence (unit: kg m^‑2^) for each cluster. The inner and outer circles represent 200 and 800 km radius, respectively.

=

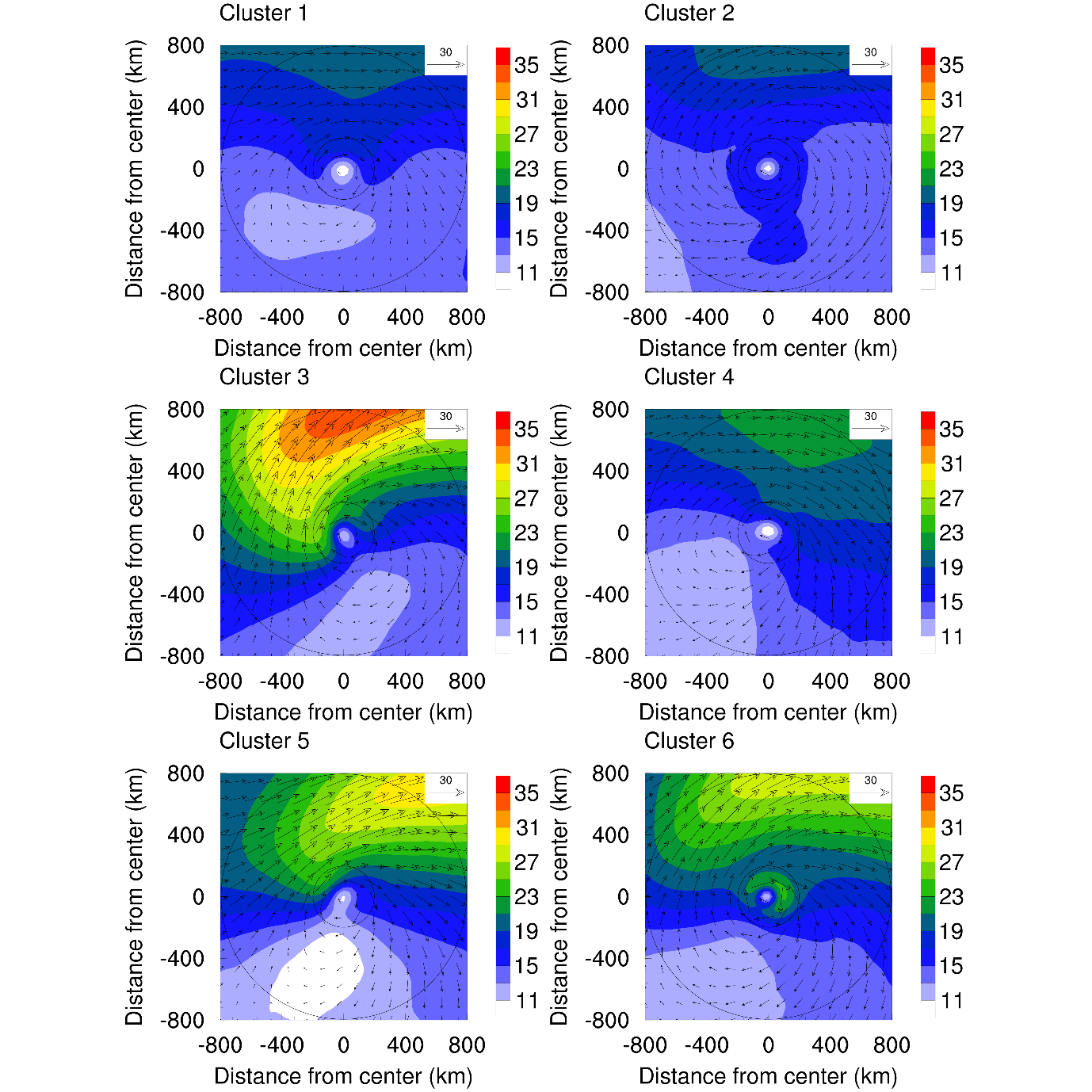


Fig. S5. Mean spatial distribution of vertical wind shear vectors and magnitudes (unit: m s^‑1^) for each cluster. The inner and outer circles represent 200 and 800 km radius, respectively.
